# Exploring the Mechanism of *Anemarrhena asphodeloides* in the Treatment of Osteoporosis Based on Network Pharmacology, Molecular Docking, and Mendelian Randomization

**DOI:** 10.1155/bmri/9283631

**Published:** 2025-09-22

**Authors:** Tao Huang, Shihao Li, Yuxin Wen, Ping Huang, Zhenwei Wu, Jun Wang, Qiong Wang

**Affiliations:** ^1^ Orthopedics, Wuhan Red Cross Hospital, Wuhan, Hubei, China; ^2^ College of Pharmacy, Hubei University of Chinese Medicine, Wuhan, Hubei, China, hbtcm.edu.cn; ^3^ Public Inspection and Testing Center of Xianning, Wuhan, Hubei, China; ^4^ College of Basic Medical Sciences, Hubei University of Chinese Medicine, Wuhan, Hubei, China, hbtcm.edu.cn

**Keywords:** *Anemarrhena asphodeloides*, Mendelian randomization, molecular docking, network pharmacology, osteoporosis

## Abstract

**Context:** Osteoporosis is a systemic bone disease characterized by reduced bone density and quality. *Anemarrhena asphodeloides* shows therapeutic potential, but its mechanisms remain unclear.

**Objective:** The aim of this study is to elucidate the potential mechanisms of *Anemarrhena asphodeloides* in the treatment of osteoporosis based on a combination of bioinformatics and experimental approaches.

**Materials and Methods:** *Anemarrhena asphodeloides*’ targets were sourced from TCMSP, while osteoporosis‐related genes came from GeneCards and OMIM. Overlapping targets (253) were analyzed via GO/KEGG. A PPI network (STRING/Cytoscape) identified core targets (degree > 5). Mendelian randomization integrated GWAS data, and molecular docking validated results.

**Results:** Two hundred fifty‐three “drug–disease” intersection targets were obtained through screening. Five positive genes were obtained by Mendelian randomization, and the molecular docking results of key targets and core active ingredients were satisfactory.

**Discussion and Conclusion:** *Anemarrhena asphodeloides* contains multiple active ingredients, which may exert therapeutic effects on osteoporosis by regulating targets such as AKR1C1, AKR1C2, ABCC1, SMO, and AKT1 and key signaling pathways like PI3K‐Akt and VEGF, thereby providing theoretical support for the clinical use of *Anemarrhena asphodeloides* in the treatment of osteoporosis.

## 1. Introduction

Osteoporosis (OP) is the most common bone disease in humans, characterized as a chronic progressive disorder with decreased bone density, abnormal bone microstructure, and increased bone fragility, exhibiting significant morbidity and mortality rates [1]. The prevalence of OP or low bone mass varies with age, gender, race, and ethnicity and increases with age [2]. Both men and women experience accelerated bone loss and a gradual decrease in bone mass during menopause [3]. With increasing age, the incidence of osteoporotic fractures also increases. These fractures are closely associated with a significant loss of quality of life, increased health care costs, and many other issues. In view of this, effective prevention and precise diagnosis of OP and its complications have become urgent public health challenges that need to be addressed [4].

A large number of clinical reports and studies have shown that traditional Chinese medicine has certain advantages in terms of compliance and efficacy in the prevention and treatment of OP. The clinical symptoms of OP are consistent with the manifestations of “pain due to obstruction,” “inability to raise the waist and spine,” “weakness in both feet,” and “inability to control the body” in traditional Chinese medicine diseases such as “bone atrophy” and “bone arthralgia.” Therefore, traditional Chinese medicine uses “bone atrophy” and “bone arthralgia” to treat OP [5]. The “Suwen” states that “the kidney governs the bones” and “the kidney governs the bone marrow of the body….” When the kidney qi is hot, the waist and spine will not be raised, the bones will become dry, and the marrow will decrease, resulting in “bone atrophy” and “kidney disorders.” The discussions such as “the essence lies in the bones” and “it fills the bones” clearly point out that kidney essence generates marrow and nourishes the bones. Modern research in traditional Chinese medicine has found that as people age, the incidence of kidney deficiency syndrome gradually increases and the content of bone minerals in human bones also gradually decreases [6]. Therefore, tonifying kidney drugs are commonly used in the clinical treatment of OP [7]. The “Suwen Xuanji Yuanbing Shi” records: “Both illness and atrophy belong to the lung metal… The lung metal is inherently dry. When it is dry, the blood supply weakens and cannot nourish the bones. This explains the close relationship between the lung and the bones. These discussions indicate that bone growth is related to the lungs and kidneys.” The Compendium of Materia Medica states: “When the kidneys are bitter and dry, it is advisable to consume pungent food to moisten them.” If the lung is bitter and rebellious, it is advisable to consume bitter food to purge it. It is known that the *Anemarrhena asphodeloides* is cold in nature, bitter and pungent in taste, and belongs to the lung, stomach, and kidney meridians. It moistens kidney dryness at the bottom and clears lung fire at the top, achieving the effect of the mutual generation of metal and water, which is beneficial to bone growth.

Traditional anti‐OP drugs have shown significant efficacy in the treatment and prevention of OP, but individual differences and possible reactions need to be paid attention to during use. This has prompted many patients to choose traditional botanicals to combat OP [8]. Traditional Chinese medicine, as an important treatment, has attracted wide attention in the research of OP in recent years [9].

*Anemarrhena asphodeloides* Bunge (AR) is a traditional medicinal plant that is the dried rhizome of *Anemarrhena asphodeloides* Bge. *Anemarrhena asphodeloides* contains flavonoids, steroidal saponins, polysaccharides, organic acids, alkaloids, phenylpropanoids, and steroidal compounds. Studies have shown that it has valuable biological activities, such as anti‐inflammatory, anti‐OP, and antioxidant [10]. Multiple studies have shown that the active ingredients in *Anemarrhena asphodeloides* play a significant role in the treatment of OP. Peng et al. found through research that Guizhi Shaoyao Zhimu decoction can effectively regulate bone metabolism indicators by inhibiting the expression of MMP‐9, TRAP5b, and CTSK while promoting ALP activity and modulating RANKL/OPG, thereby enhancing bone density in rats. This decoction can alleviate the thinning of the growth plate at the distal femoral epiphysis, increase the number and width of trabeculae, and significantly improve their structural connectivity [11]. Anemarsaponin A‐III alleviates diabetic OP by inhibiting the RAGE/MAPK signaling pathway, downregulating AGE‐induced levels of IL‐1*β*, IL‐6, and TNF‐*α*, and upregulating levels of alkaline phosphatase and osteocalcin [12]. [13]) demonstrated that Anemarsaponin B‐II activates autophagy by inhibiting the mTOR/NF*κ*B signaling pathway in osteoblasts, upregulating the expression of Beclin1, thereby alleviating OP induced by hyperglycemia. [14]) found through research that Anemarsaponin B‐II combined with icariin can inhibit osteoclast differentiation, promote osteoblast differentiation, and maintain the balance between osteoclasts and osteoblasts. Other studies have shown that the polysaccharide components in *Anemarrhena asphodeloides* play an important role in the treatment of OP [15].

Network pharmacology is an emerging discipline based on systems biology theory, which uses biological system network analysis and multitarget drug molecule design to select specific signal nodes [16]. Through the constructed drug–disease target network diagram, the mechanism of action and key genes of the drug can be clearly understood. Molecular docking is a mature computer‐based structural technique widely used in new drug development. It can discover novel compounds of medical significance and infer ligand–target interactions at the molecular level or describe structure–activity relationships [17]. Mendelian randomization uses genetic variation as a natural experiment to explore the causal link between potentially modifiable exposure factors and outcome factors in the observational data [18]. In this study, by using network pharmacology, Mendelian randomization, and molecular docking methods to explore the effective ingredients and potential targets of *Anemarrhena asphodeloides* in the treatment of OP, TCM research can break through the bottleneck of traditional research and improve its research efficiency and accuracy. This integrated approach can not only reveal the multitarget and multipathway mechanism of TCM but also verify its causal relationship and molecular‐level interaction, providing more powerful scientific support for the clinical application of TCM, drug development, and personalized treatment. To verify the efficacy of *Anemarrhena asphodeloides* in the treatment of OP, we first established the *Anemarrhena asphodeloides*–target–OP regulatory network through network pharmacological analysis. Then, we conducted Mendelian randomization analysis using OP data from OpenGWAS and the intersecting targets to identify significant single‐nucleotide polymorphisms (SNPs) and genes. Finally, we validated the findings through molecular docking.

## 2. Materials and Methods

### 2.1. Animal Origin

SPF‐grade female SD rats, 10 weeks old, weighing 260–280 g, were purchased from the Animal Center of China Three Gorges University, with License Number SCXK (E) 2022‐0012. The animal experiments were conducted under the guidance of the Ethics Committee of Hubei University of Chinese Medicine (Ethics Review Number HUCMS202205005) and the National Laboratory Animal Use Law (China), in accordance with the principles of the use and care of laboratory animals. The rats were housed in the Experimental Animal Center of Hubei University of Chinese Medicine.

### 2.2. Source of Medicinal Materials

The *Anemarrhena asphodeloides* slices were purchased from Hubei Tianji Pharmaceutical Co. Ltd., Batch Number 20211101.

### 2.3. Establishment of OP Models

Thirty‐six rats were randomly divided into the sham operation group (sham, *n* = 9) and the model group (model, *n* = 27). The model group underwent oophorectomy to remove both ovaries. The specific methods were as follows: After general anesthesia was administered to rats by intraperitoneal injection of 3% pentobarbiturate sodium (40 mg/kg), the rats were placed on the operating table in a supine position, and their limbs were fixed. The skin of the surgical area was disinfected with medical alcohol. A small incision was made at the midline of the lower abdomen of the rat. The skin, subcutaneous tissue, abdominal muscles, and peritoneum were cut successively to expose the ovaries and surrounding structures in the abdominal cavity. After the ovaries were completely removed with surgical scissors, the residual ends of the ovaries were ligated with silk thread. After checking that there was no bleeding or other abnormalities in the abdominal cavity, sutures were applied to the abdominal muscles, peritoneum, subcutaneous tissue, and skin layer by layer. The surgical process for rats in the sham operation group was the same down to the step of exposing the ovaries, but no oophorectomy was performed. Only the incision was sutured as a control. For 3 consecutive days after the operation, 40,000 U/day penicillin was intramuscularly injected into the thigh area to prevent wound infection. The healing of the skin incision, mental state, and feeding and water intake of the rats were closely observed. After 6 weeks of conventional feeding, three rats in each of the sham operation group and the model group were sacrificed under random anesthesia. The right femur was removed. A microcomputed tomography scanner was used to verify whether the establishment of the OP model was successful. The micro‐CT results showed that the modeling was successful.

### 2.4. Drug Administration Method

Fourteen weeks after modeling, 24 rats in the model group were randomly divided into the ovariectomized group (OVX, *n* = 8), the ovariectomized + Anemarrhena group (AR, *n* = 8), and the ovariectomized + estradiol valerate group (EV, *n* = 8). The ovariectomy + *Anemarrhena* group was given *Anemarrhena* decoction with a mass concentration of 2.16 g/kg. The ovariectomy + estradiol valerate group was given estradiol valerate tablet solution with a mass concentration of 0.09 mg/kg. The ovariectomy group and the sham operation group were orally administered with the same volume of pure water. Rats in each group were administered by gavage once a day and weighed once a week for a total of 14 weeks. The abovementioned drug dosages have all been normalized based on the body surface area and converted according to the clinical dosages stipulated in the pharmacopeia.

### 2.5. Anatomy and Sampling

After rats were anesthetized with 3% pentobarbital sodium, blood was collected from the abdominal aorta. After the blood collection was completed, the mice were fixed on the anatomical table. The abdominal part of the rats was cut open with surgical scissors to collect the uterine tissue and weighed. The left leg was cut open; the femoral tissue was stripped off and then quickly frozen in liquid nitrogen and stored in a low‐temperature freezer at −80°C for testing. The blood samples were left to stand at 4°C for 2 h and then centrifuged using a low‐temperature centrifuge (3000 rpm). The serum was taken out and stored at −20°C for testing.

### 2.6. Identification of Intersection Target Genes of Drugs and Diseases

The active ingredients and drug targets of *Anemarrhena asphodeloides* were obtained from the TCMSP database (https://old.tcmsp-e.com/tcmsp), with parameters set at OB > 30*%* and DL > 0.18 for selecting active ingredients (Table S1). The drug targets related to the active ingredients were obtained using a Perl script. The full names of the genes and their corresponding symbols were retrieved through the UniProt database using a Perl script. OP‐related genes were obtained from the Gene Map in the GeneCards database (https://www.genecards.org/) and the OMIM database (https://www.omim.org/help/about). The genes obtained from the above sources were intersected with the target genes of *Anemarrhena asphodeloides* using R 4.4.1 software, and the resulting genes were used as target genes for subsequent analysis.

### 2.7. Screening of Core Target Genes Through Protein–Protein Interaction (PPI) Analysis

The target genes obtained from the intersection were imported into the STRING platform for analysis, with the species set to *Homo sapiens* and the minimum interaction score set to 0.9, while other parameters were kept at default settings. The results obtained from the analysis were then imported into Cytoscape (Version 3.10.2), and a node threshold of degree centrality > 5 was set to identify the core target genes.

### 2.8. GO and KEGG Enrichment Analysis

In order to further study the biological processes involved in these core target genes, clusterProfiler in R 4.4.1 was used for enrichment analysis of molecular functions, cell components, and biological processes, as well as signaling pathway analysis, and the significance standard was set as *p* < 0.05.

### 2.9. Mendelian Randomization Analysis

Based on the screening results of the drug–disease intersection targets as the exposure factor, OP was entered into the OpenGWAS database (https://gwas.mrcieu.ac.uk/) to obtain GWAS data related to OP as the outcome factor. To avoid the influence of linkage disequilibrium on the experimental results, the selected SNPs adhered to the criterion of *p* < 5 × 10^−8^, with thresholds set at *r* ^2^ = 0.001 and genetic distance = 10,000 kb to ensure the independence of the instruments. To make the results more convincing, the significance threshold of *F* > 10 was chosen when screening instrumental variable SNPs. The instrumental variables related to the exposure factor should be identified according to the following criteria: (1) Correlation assumption: The selected SNPs of the instrumental variables must be strongly correlated with the exposure factor (intersection targets). (2) Independence assumption: The instrumental variables must be unrelated to potential confounding factors between the exposure and the outcome. (3) Exclusion assumption: The influence of the instrumental variables on the outcome should only be through the exposure factor and not through other pathways [19].

### 2.10. Sensitivity Analysis

Sensitivity analysis comprised tests for horizontal pleiotropy, heterogeneity, and leave‐one‐out analysis. The MR‐Egger regression analysis was applied to assess the presence of horizontal pleiotropy, and a significant intercept in MR‐Egger analysis indicated horizontal pleiotropy. Cochran’s *Q* test assessed SNP heterogeneity; a statistically significant Cochran’s *Q* statistic (*p* ≤ 0.05) indicated notable heterogeneity in the analysis outcomes. The MR pleiotropy residual sum and outlier (MR‐PRESSO) test identified outliers, and if detected, these outliers were removed, and the remaining instrumental variables were reanalyzed. A leave‐one‐out approach was implemented to evaluate if the statistical significance of the results was influenced by the inclusion of particular SNPs. The correlation between intersection target and the likelihood of OP onset was quantified using the beta value alongside its 95% confidence interval (CI). A *p* value of 0.05 or lower was interpreted as suggestive of a possible causal link.

### 2.11. Molecular Docking Validation

Select the protein encoded by the intersecting target gene as the receptor and the active ingredient of *Anemarrhena asphodeloides* as the ligand for molecular docking. The protein encoded by the intersecting target gene is obtained from the PDB database (https://www.rcsb.org/), and the molecular structure of the active ingredient of *Anemarrhena asphodeloides* is obtained from the PubChem database (https://pubchem.ncbi.nlm.nih.gov). The above 3D structures were all processed using AutoDock Vina 1.0 to remove water molecules, add hydrogen atoms, and assign charges and then converted into pdbqt files. Finally, after molecular docking, PyMOL 2.3.0 was used to create a schematic diagram for visualization.

### 2.12. Western Blot Analysis

A 50‐mg sample was taken from the left femoral tissue, and proteins were extracted using RIPA lysis buffer. Then, the protein concentration was determined by BCA method. After sample loading, electrophoresis, membrane transfer, and closure were performed successively. Then, primary antibodies for AKR1C1 (1:1000), AKR1C2 (1:1000), ABCC1 (1:1000), AKT1 (1:1000), Smoothened (SMO) (1:1000), and GAPDH (1:10000) were added, and the membranes were incubated overnight at 4°C. After washing, secondary antibodies (1:5000) were added for incubation, and the membranes were washed again. Afterwards, ultrasensitive ECL luminescent solution was used for color development, and images were captured and measured using an imaging device. Densitometric analysis was performed using ImageJ2x software (National Institutes of Health, United States).

### 2.13. Statistical Analysis

Data were statistically analyzed and plotted using GraphPad Prism 10.1.2, except for labels annotated using analysis software. The results are presented as mean ± standard deviation (SD). Comparisons between two groups and among multiple groups were performed using *t*‐tests and one‐way analysis of variance (ANOVA), respectively. Statistical significance was defined as *p* < 0.05.

## 3. Results

### 3.1. Intersection Target Genes of *Anemarrhena asphodeloides* and OP

The 25 components in *Anemarrhena asphodeloides* yielded a total of 398 potential targets, which were intersected with 6435 OP‐related targets to form 253 overlapping targets, as shown in Figure 1a. To further investigate the mechanism of *Anemarrhena asphodeloides* in the treatment of OP, we constructed a composite target network as illustrated in Figure 1b, containing 280 nodes and 1663 edges.

Figure 1(a) A Venn diagram of the intersecting gene targets between *Anemarrhena asphodeloides* and OP. (b) A network diagram of the active components of *Anemarrhena asphodeloides* and OP genes. (c) A PPI network diagram of *Anemarrhena asphodeloides* and OP genes. (d) The core target genes selected from the PPI network with a degree value > 5. (e, f) The bubble chart and circular Manhattan plot for GO and KEGG analyses.

**Figure:**
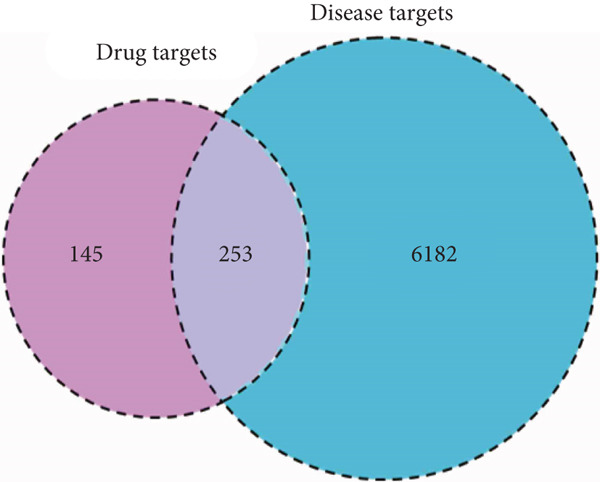
(a)

**Figure:**
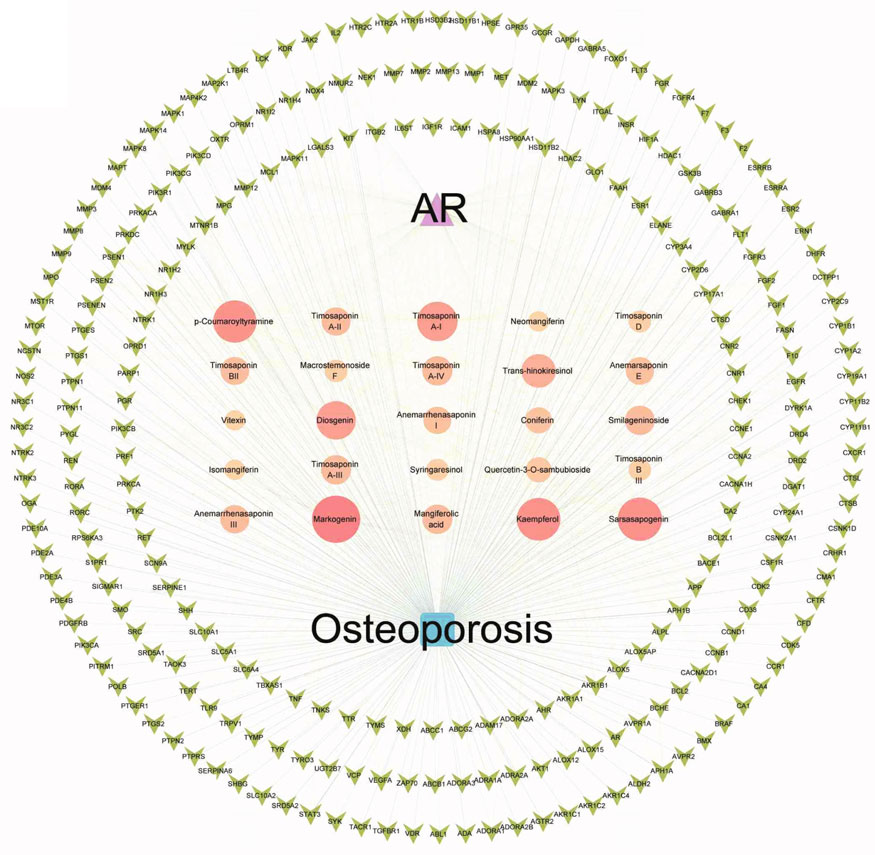
(b)

**Figure:**
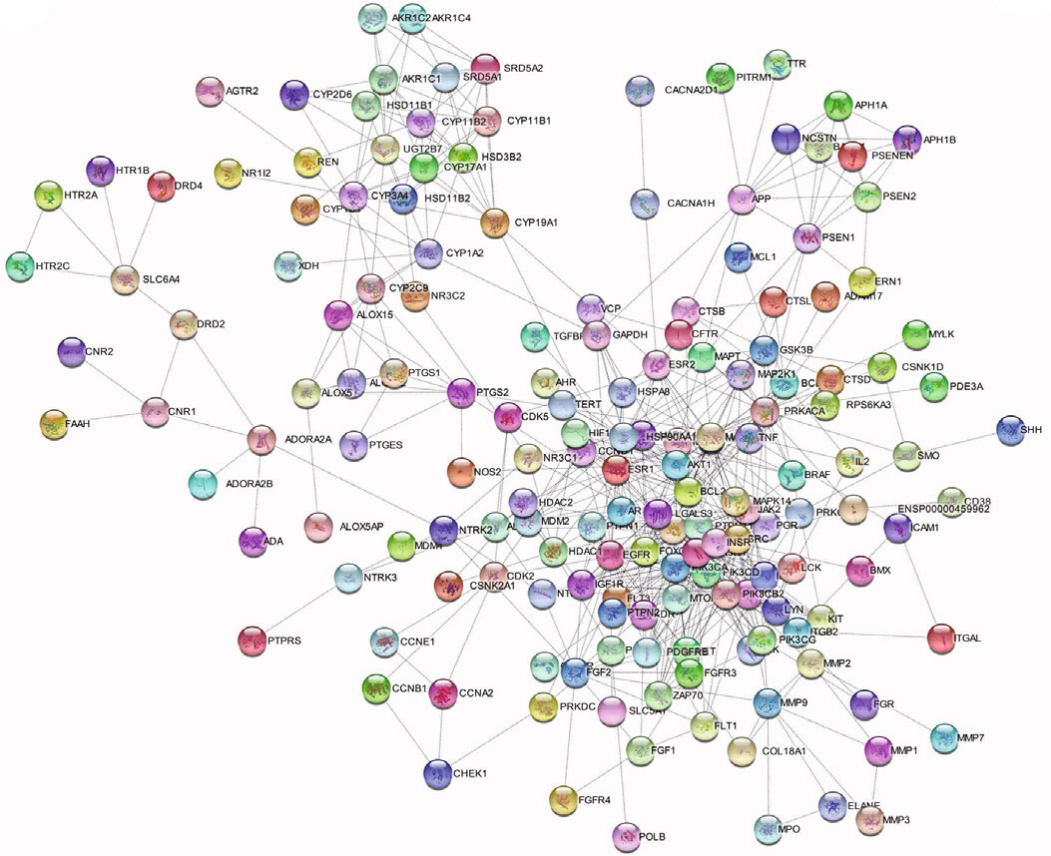
(c)

**Figure:**
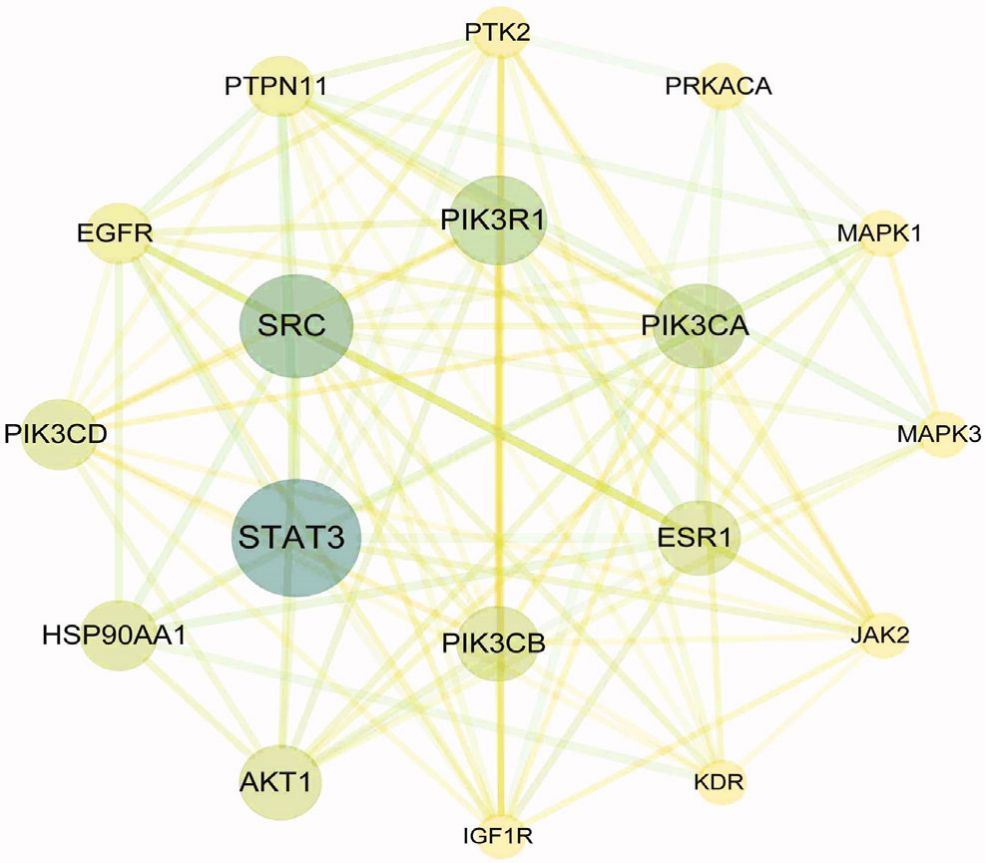
(d)

**Figure:**
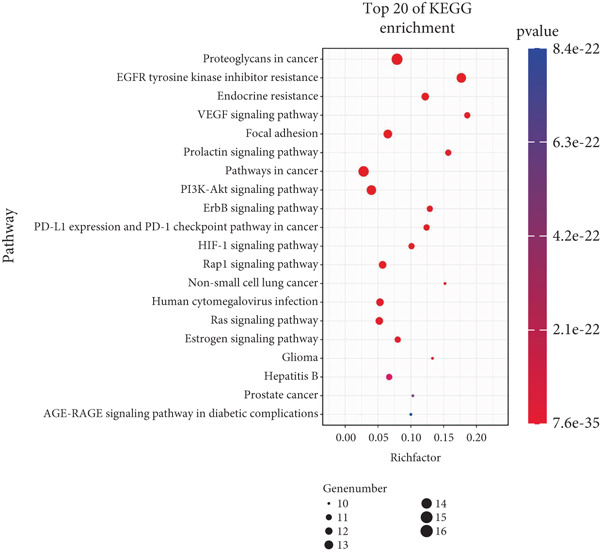
(e)

**Figure:**
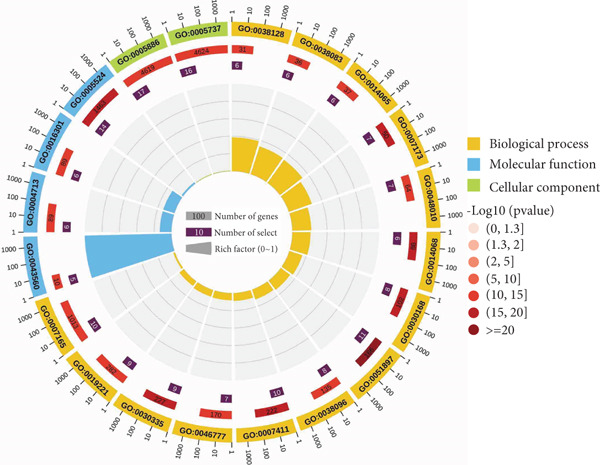
(f)

### 3.2. Construction of PPI Network for *Anemarrhena asphodeloides* and OP Target Genes

The intersection target genes were processed for PPI using the STRING platform. As shown in the PPI network in Figure 1c, *Anemarrhena asphodeloides* can exert its therapeutic effects through multiple targets. Targets with a degree centrality > 5 were selected as core targets to further construct the network diagram shown in Figure 1d.

### 3.3. GO and KEGG Enrichment Analysis

GO enrichment analysis (Figure 1e) showed that biological processes were mainly involved in the ERBB2 signaling pathway (GO:0038128), peptide tyrosine autophosphorylation (GO:0038083), and phosphatidylinositol 3‐kinase signaling (GO:0014065), among others. The molecular functions are mainly manifested in insulin receptor substrate binding (GO:0043560), protein tyrosine kinase activity (GO:0004713), and ATP binding (GO:0005524), among others. The cellular components are primarily located in the plasma membrane (GO:0005886) and cytoplasm (GO:0005737). KEGG pathway analysis (Figure 1f) showed that *Anemarrhena asphodeloides* may regulate the PI3K‐Akt signaling pathway, HIF‐1 signaling pathway, and VEGF signaling pathway. These pathways are related to metabolism, inflammation, and immune response, suggesting that *Anemarrhena asphodeloides* has a therapeutic effect on OP through these pathways.

### 3.4. Results of Mendelian Randomization Analysis

**Figure 2:**
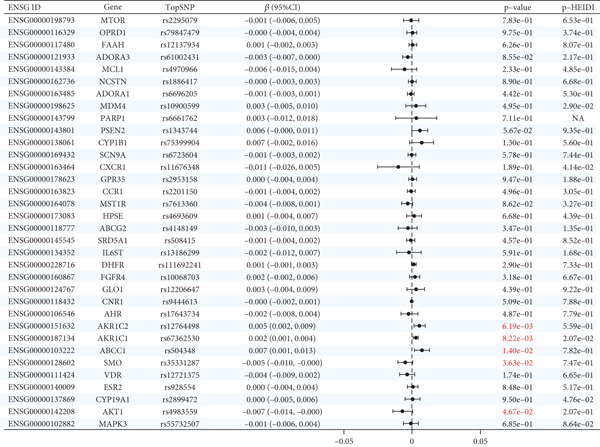
Summary of the MR significance results of the intersecting target genes with OP, with the highlighted red indicating positive results with *p* < 0.05.

The analysis results showed that there were five genes consistent with *p* < 0.05, which had research significance, as shown in Figure 2. Among them, AKR1C1, AKR1C2, and ABCC1 exhibited consistent trends in their causal effect estimates on OP, indicating they are risk factors. In contrast, SMO and AKT1 showed opposite trends in their causal effect estimates on OP, suggesting they are protective factors.

### 3.5. Sensitivity Analysis

The presence and outcome of horizontal pleiotropy among SNPs were evaluated by MR‐Egger regression analysis. For AKR1C1 (intercept *p* = 0.603), AKR1C2 (intercept *p* = 0.676), ABCC1 (intercept *p* = 0.754), SMO (intercept *p* = 0.626), and AKT1 (intercept *p* = 0.164), all MR‐Egger regression intercepts were greater than 0.05, indicating the lack of horizontal pleiotropy between SNPs and outcomes. Cochran’s *Q* test results for AKR1C1, AKR1C2, ABCC1, SMO, and AKT1 in both IVW and MR‐Egger analyses showed *Q*_pval > 0.05, confirming the absence of heterogeneity in the selected SNPs [20]. The results indicate the robustness of the MR analysis, as shown in Table 1. Figure 3 shows that AKR1C1, AKR1C2, and ABCC1 are risk factors for OP, while SMO and AKT1 are protective factors for OP.

**Table 1:** Sensitivity analysis of the causal relationship between positive target genes and osteoporosis.

**Exposure factor**	**Heterogeneity**			**Pleiotropy**	
	**Method**	**Q**	**p**	**Egger intercept**	**p**
AKR1C1	IVW	2	0.772	4.722e − 04	0.603
AKR1C2	IVW	4	0.470	3.283e − 04	0.676
ABCC1	IVW	3	0.492	3.810e − 04	0.754
SMO	IVW	2	0.800	1.514e − 04	0.626
AKT1	IVW	6	0.305	2.474e − 04	0.164

Figure 3(a) The main impact of AKR1C1 on OP. (b) The main impact of AKR1C2 on OP. (c) The main impact of ABCC1 on OP. (d) The main impact of SMO on OP. (e) The main impact of AKT1 on OP.

**Figure:**
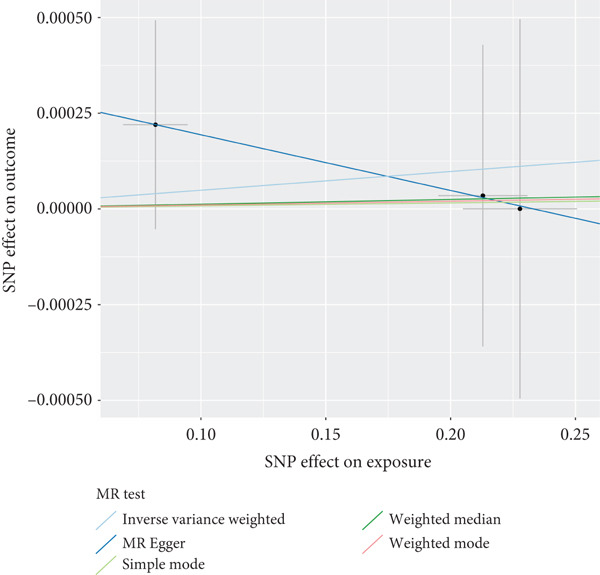
(a)

**Figure:**
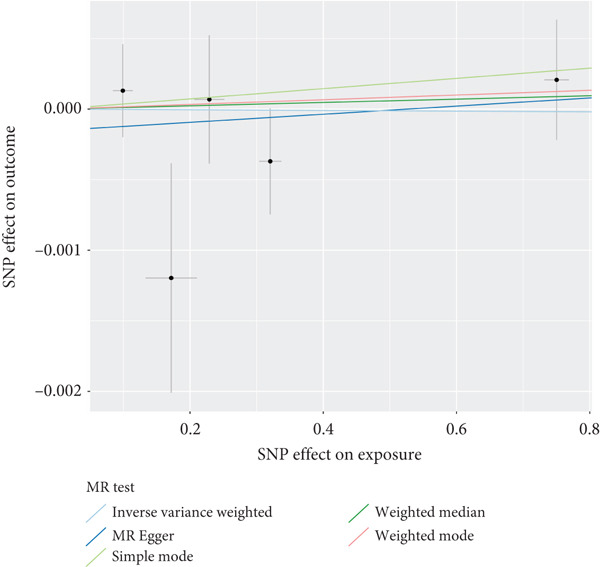
(b)

**Figure:**
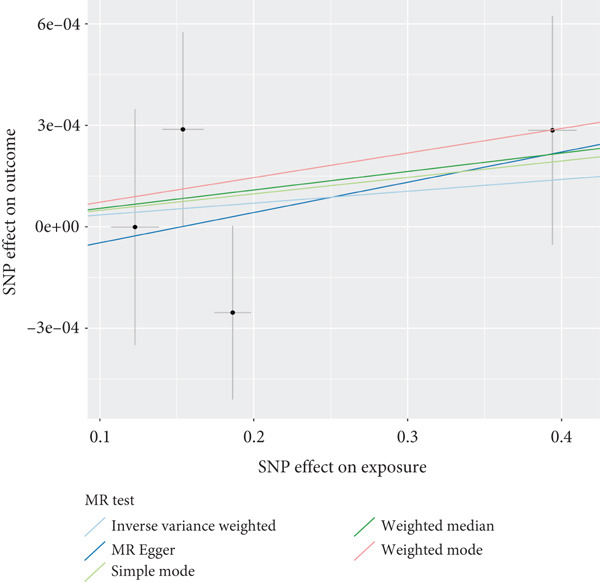
(c)

**Figure:**
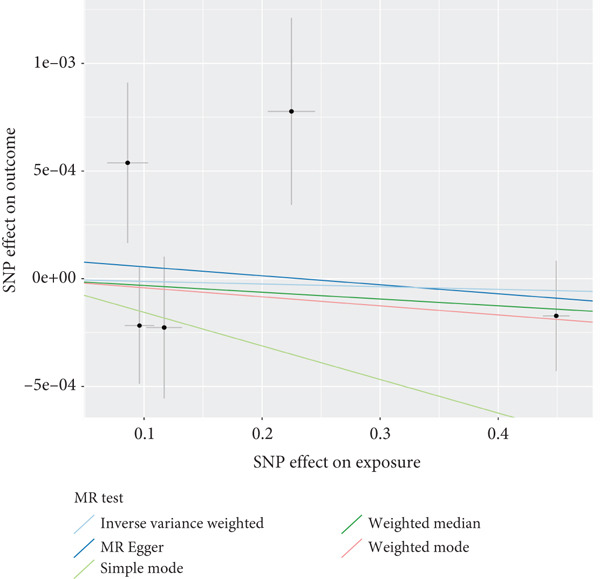
(d)

**Figure:**
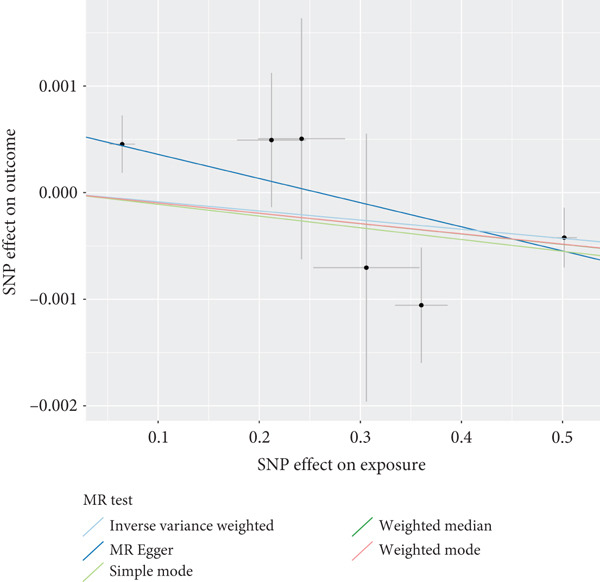
(e)

### 3.6. Colocalization Analysis Results

As shown in Figure 4, the colocalization analysis results indicate that AKR1C1, AKR1C2, ABCC1, SMO, and AKT1 share common genetic SNPs with OP, but the posterior probability does not reach the threshold for colocalization.

Figure 4(a–e) The lead SNP with the highest posterior probability according to color, with other SNPs colored based on their LD *r* ^2^ with the lead SNP.

**Figure:**
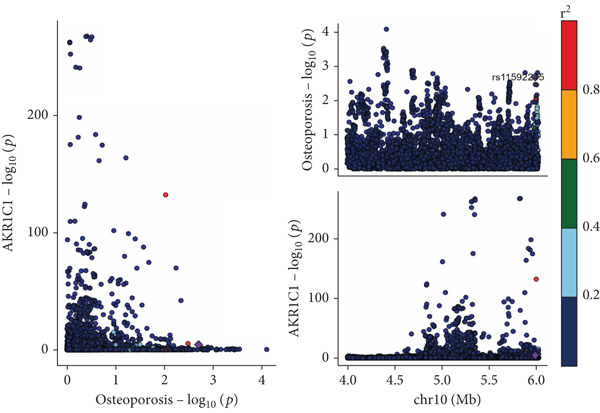
(a)

**Figure:**
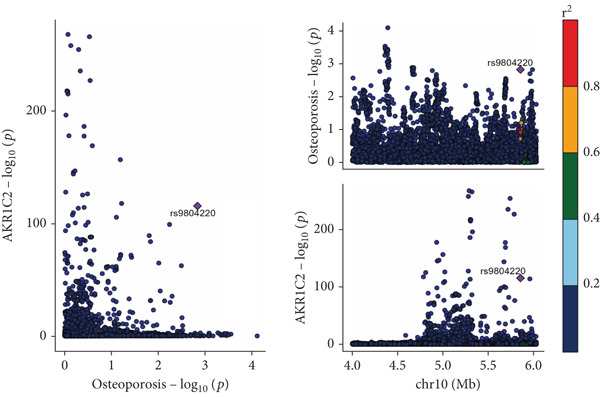
(b)

**Figure:**
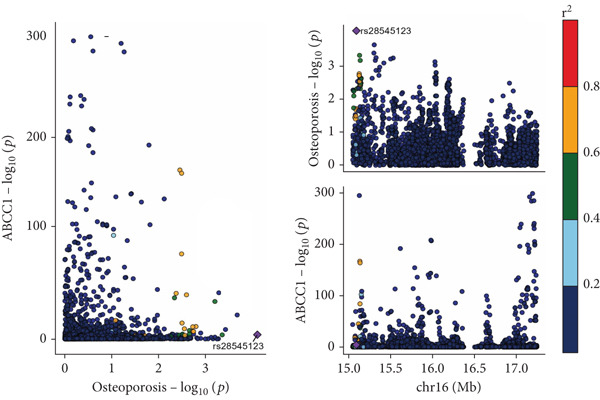
(c)

**Figure:**
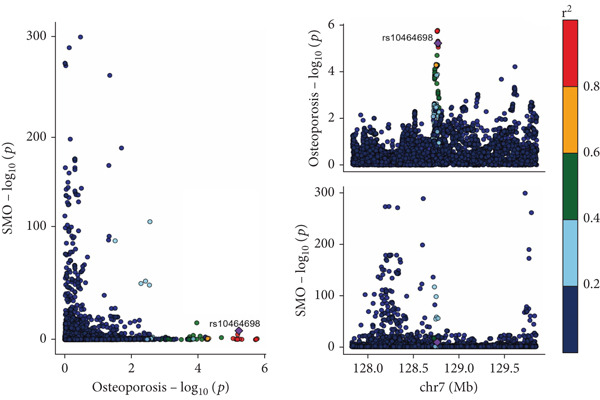
(d)

**Figure:**
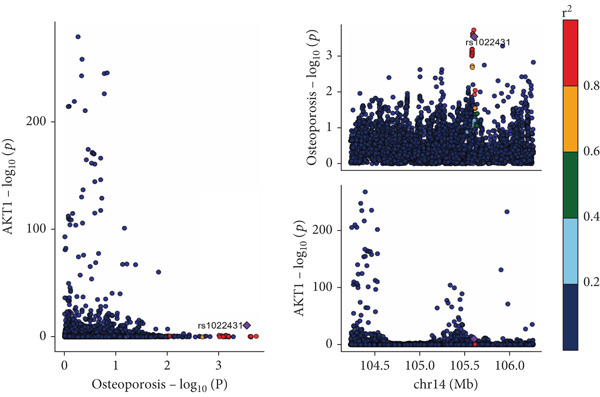
(e)

### 3.7. The Result of Molecular Docking

AutoDock Vina 1.0 was used to perform molecular docking between key target proteins and important active ingredients (see Table 2). The results showed that the binding energies of all dockings were below −5.0 kcal/mol, indicating strong binding affinity between the active components of *Anemarrhena asphodeloides* and the targets for the treatment of OP. The specific molecular docking mode is shown in Figure 5.

**Table 2:** Molecular docking results of key pharmacological components of *Anemarrhena asphodeloides* with target proteins.

**Drug active ingredient**	**Binding energy (kJ/mol)**				
	**AKR1C1 (1MRQ)**	**AKR1C2 (4XO6)**	**ABCC1 (2CBZ)**	**SMO (4JKV)**	**AKT1 (1UNQ)**
Sarsasapogenin	−12.2	−10.3	−7.7	−10.1	−7.5
Timosaponin A‐I	−11.8	−10	−8.3	−10.9	−8.1
Timosaponin A‐III	−9.6	−9.2	−8.8	−10.1	−8.4
Markogenin	−11.2	−10.8	−8.1	−10.2	−7.9
Neomangiferin	−9.6	−11	−7.5	−8.6	−7
Anemarrhenasaponin III	−8.5	−9	−8.6	−10.1	−8
Timosaponin B‐III	−8.7	−9.8	−8.6	−9.4	−7.7
Timosaponin B‐II	−9.7	−9	−7.7	−8.7	−7.8
Isomangiferin	−8.2	−10.2	−6.6	−8.6	−6.4
Anemarsaponin E	−9.2	−9.8	−9.1	−10.5	−7.6

Figure 5(a) Timosaponin A‐I SMO. (b) Timosaponin A‐I AKR1C1. (c) Sarsasapogenin AKR1C1. (d) Timosaponin A‐III AKT1. (e) Timosaponin E ABCC1. (f) Neomangiferin AKR1C2.

**Figure:**
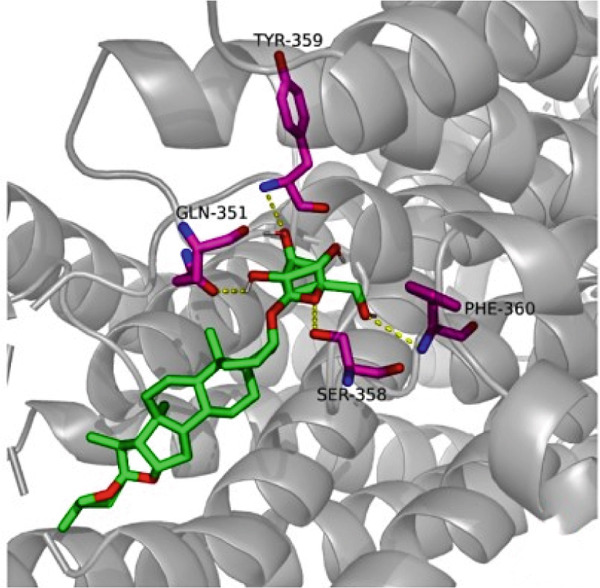
(a)

**Figure:**
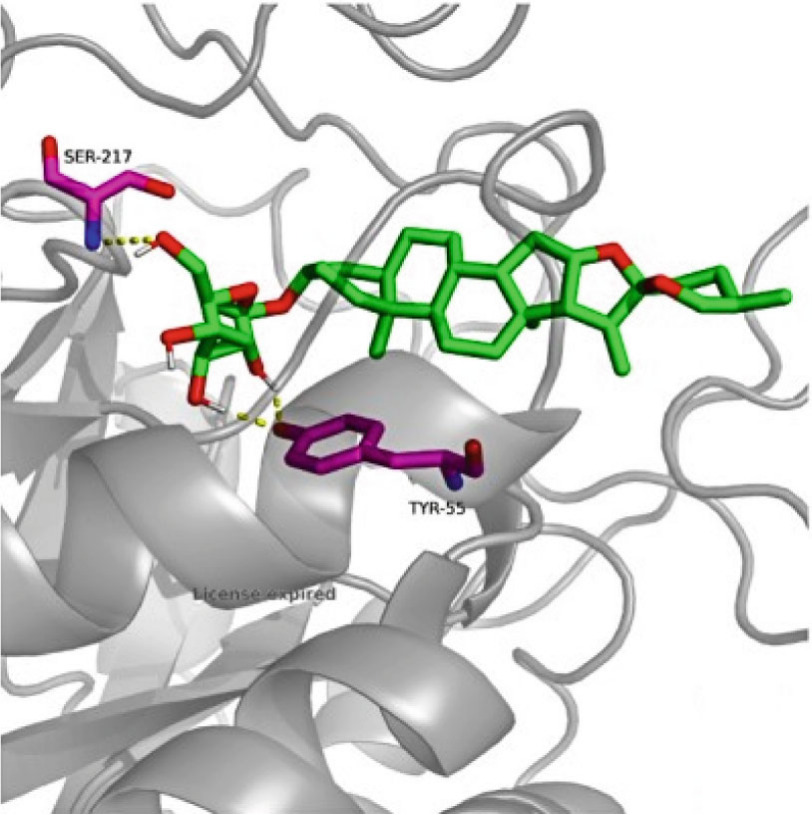
(b)

**Figure:**
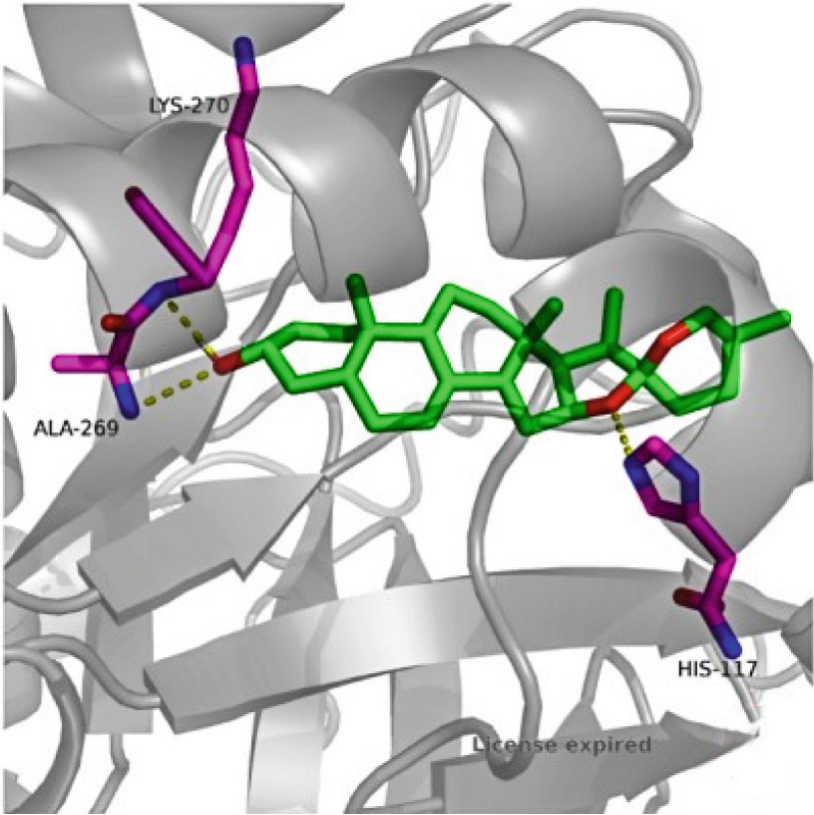
(c)

**Figure:**
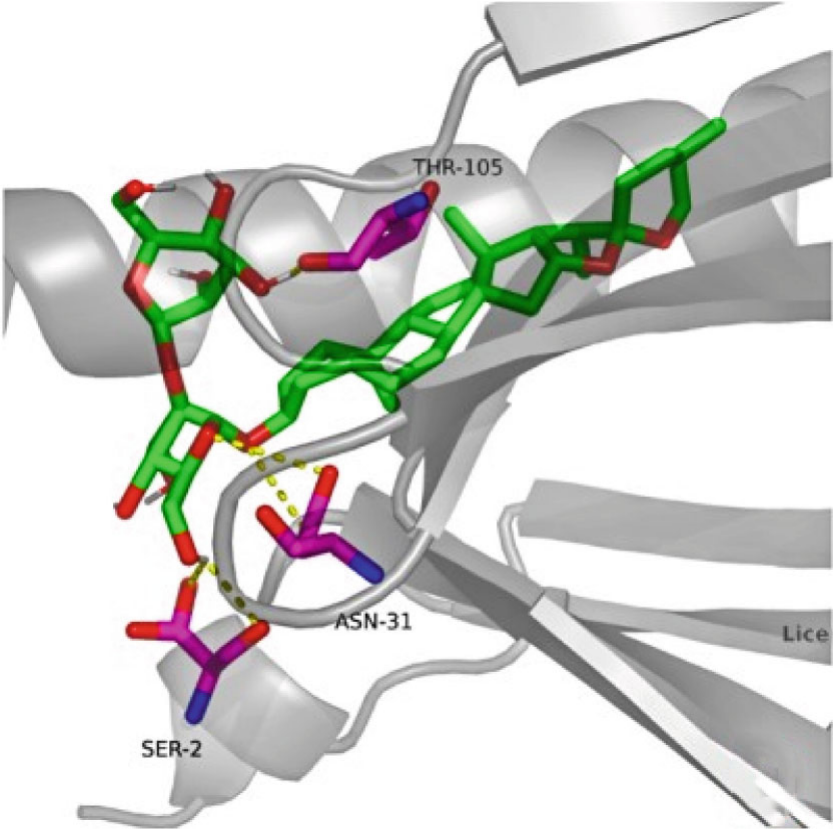
(d)

**Figure:**
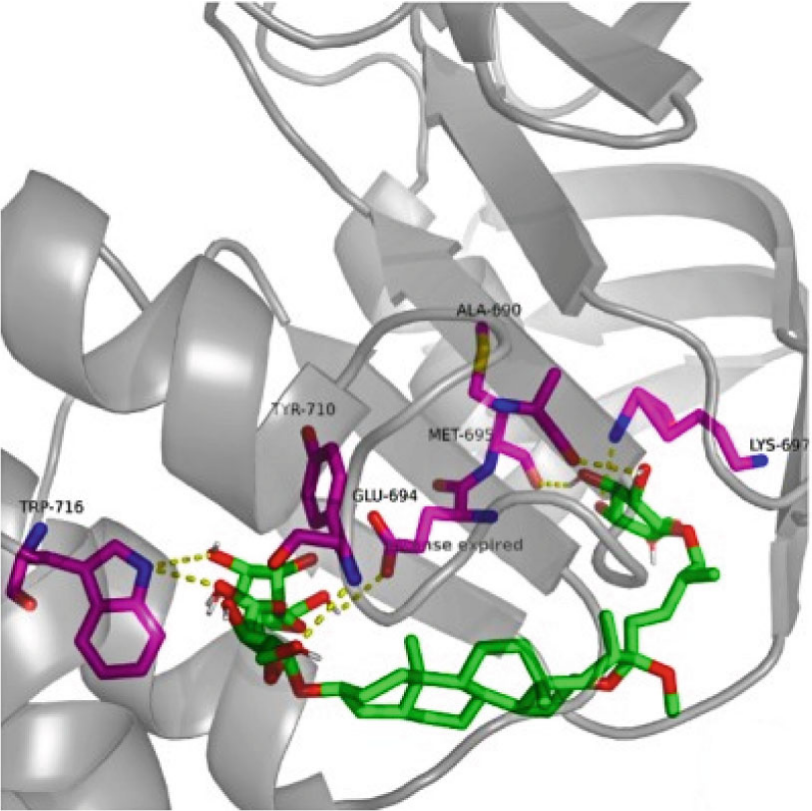
(e)

**Figure:**
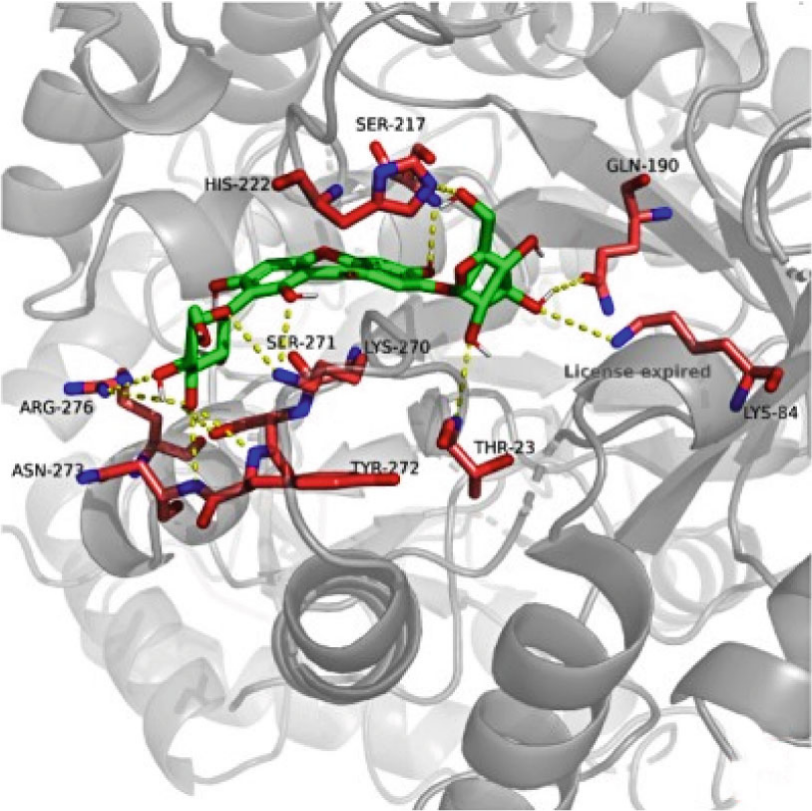
(f)

### 3.8. Results of Western Blot Analysis

In order to further confirm whether the above targets were involved in the therapeutic effect of *Anemarrhena asphodeloides* on OP, we used western blot to detect the expression of relevant targets in femoral tissue. As shown in Figure 6, *Anemarrhena asphodeloides* treatment significantly downregulated the expression of AKR1C1, AKR1C2, and ABCC1 (compared with the Mod group, *p* < 0.01) and upregulated the expression of AKT1 and SMO (compared with the Mod group, *p* < 0.01). These results suggest that these targets may be potential therapeutic targets for the role of *Anemarrhena asphodeloides* in the treatment of OP.

Figure 6(a) Protein expression of key targets in femoral tissue. (b) The quantitative result of ABCC1. (c) The quantitative result of AKR1C2. (d) The quantitative result of AKT1. (e) The quantitative result of AKR1C1. (f) The quantitative result of SMO. Data is shown as mean ± SD (*n* = 3).  ^∗^ *p* < 0.05 and  ^∗∗^ *p* < 0.01 versus Con group;  ^∗^ *p* < 0.05 and  ^∗∗^ *p* < 0.01 versus Mod group.

**Figure:**
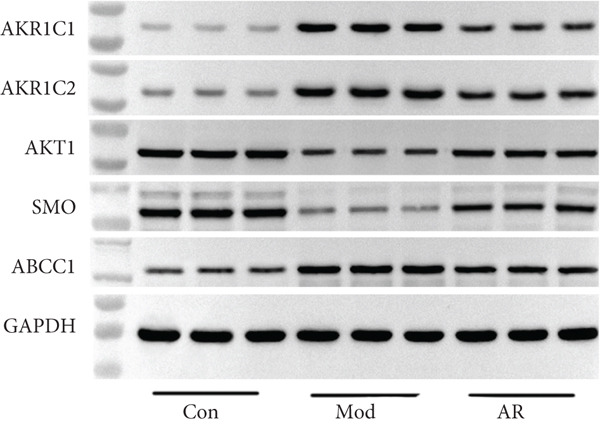
(a)

**Figure:**
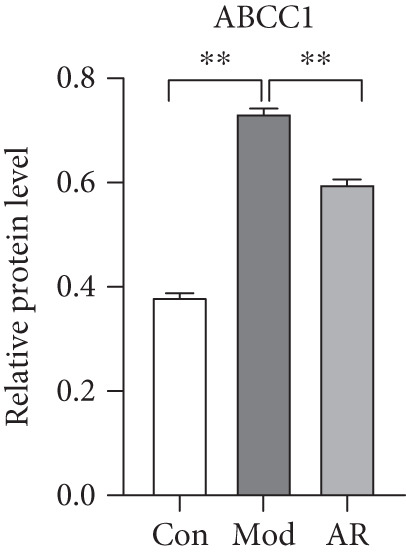
(b)

**Figure:**
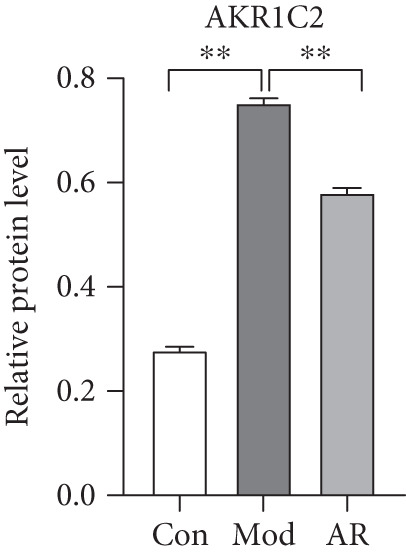
(c)

**Figure:**
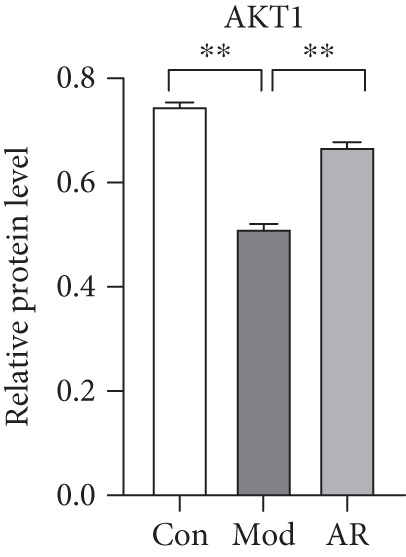
(d)

**Figure:**
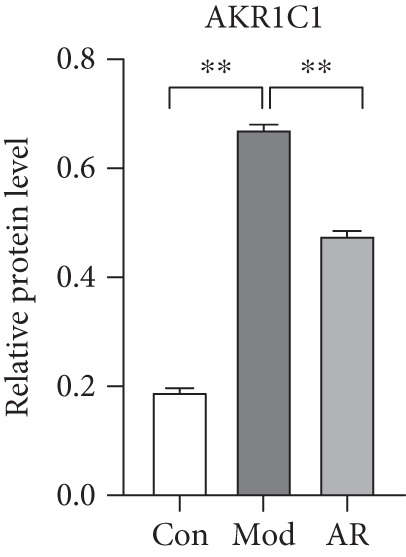
(e)

**Figure:**
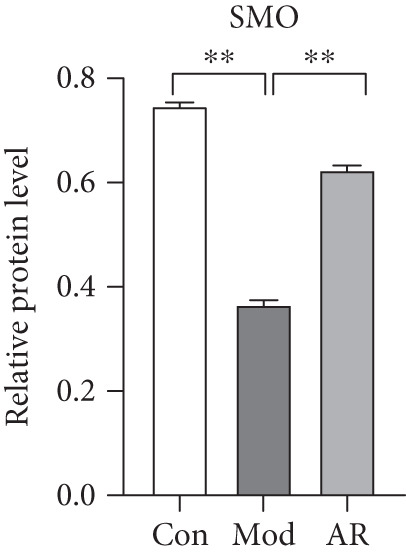
(f)

## 4. Discussion

According to the traditional Chinese medicine theory, the kidneys store essence and govern the bones and marrow, and deficiency of kidney yin is a significant cause of OP. OP is often regarded as a manifestation of “kidney deficiency” or “deficiency of liver and kidney.” The theory of “the kidneys governing the bones and generating marrow” emphasizes the important role of the kidneys in the processes of bone formation, healing, and repair [21]. Rhizoma Anemarrhenae, a commonly used herbal medicine in traditional Chinese medicine, belongs to the lung, stomach, and kidney meridians, and its medicinal value lies in clearing heat, purging fire, nourishing yin, and tonifying the kidneys [22]. Studies have shown that the active substances contained in *Anemarrhena asphodeloides* can effectively alleviate OP, primarily through the mechanisms of nourishing yin and tonifying the kidneys, as well as regulating the balance of bone metabolism [23]. This study explored the potential targets and pathways of *Anemarrhena asphodeloides* in the treatment of OP based on network pharmacology, Mendelian randomization, and molecular docking techniques, providing certain data support for the clinical use of *Anemarrhena asphodeloides* in the treatment of OP.

AKT, which refers to protein kinase B, includes three isoforms: AKT1, AKT2, and AKT3, playing a crucial regulatory role in the PI3K‐Akt signaling pathway [24]. This study focuses on the role of AKT1 in the treatment of OP. Studies have indicated that kaempferol, an effective component in *Anemarrhena asphodeloides*, may tightly bind to AKT1 through hydrogen bonds, pi‐alkyl interactions, etc. By activating the PI3K‐Akt pathway, it promotes the phosphorylated expression of AKT1, thereby achieving the therapeutic effect on OP [25–27]. [28]) found that vitexin in *Anemarrhena asphodeloides* can activate the PI3K‐Akt pathway under hypoxic conditions and upregulate the level of AKT1 to promote the differentiation of osteoblasts, thereby preventing and treating OP. Moreover, the Mendelian randomization results indicated that the trend of AKT1 was opposite to that of OP. As a protective factor, an increase in AKT1 content would reduce the risk of OP. Our WB experiment supported this result. Therefore, we can speculate that the active ingredients in *Anemarrhena asphodeloides* may regulate the expression of AKT1 protein and upregulate the level of AKT1 to prevent and treat OP.

AKR1C1 and AKR1C2 belong to the aldehyde–ketone reductase family and are involved in various physiological processes, including the metabolism of steroid hormones, drug metabolism, and lipid metabolism. The occurrence of OP is closely related to changes in hormone levels, particularly changes in estrogen and androgen [29]. Nie et al. [30] found through research that AKR1C2 can reduce highly active dihydrotestosterone in the prostate to less active 3*α*‐diol, thereby reducing androgen levels. The decrease in androgen levels is one of the important causes of OP. AKR1C2 plays a role in androgen metabolism, and its overexpression may lead to changes in gene expression, resulting in increased androgen breakdown or inactivation, which will lead to decreased androgen levels [31]. [32]) found that AKR1C1 can act as a negative regulator of osteogenic differentiation by targeting the progesterone receptor, and estrogen deficiency (such as in postmenopausal women) increases the risk of OP. AKR1C2 leads to a decrease in androgen levels, while AKR1C1 leads to a decrease in estrogen levels. Therefore, it can be speculated that the active ingredients in *Anemarrhena asphodeloides* may prevent and treat OP by inhibiting the expression of AKR1C1 and AKR1C2 in the body.

SMO is a protein that plays a crucial role in cell signal transduction, primarily associated with the Hedgehog signaling pathway, which has important functions in skeletal development and bone metabolism [33]. Studies have shown that the Hedgehog signaling pathway coordinates endochondral ossification and balances osteoblast and osteoclast activation to maintain homeostasis in vertebrates. Moreover, the activation of this pathway is transduced through the SMO gene and downstream effectors [34]. Another study has found that the loss of SMO in osteoclasts can alleviate the aging phenotype characterized by low trabecular bone mass, and the blockade of the Hh signaling pathway in osteoclasts caused by SMO exerts a protective effect against bone loss [35]. The Hh signaling pathway is activated during osteogenic differentiation of adult bones, but its activity decreases as individuals age. If the SMO gene is deleted, the Hh signaling pathway becomes inactivated, which leads to a decrease in bone formation and ultimately results in OP [36]. Diosgenin contained in the *Anemarrhena asphodeloides* can promote the activation of the Hedgehog signaling pathway, thereby enhancing osteogenic differentiation of rat bone marrow stem cells. After the treatment of rat bone marrow stem cells with diosgenin, the content of SMO in stem cells showed an upward trend, and this upward trend was positively correlated with the increase of diosgenin concentration [37]. Moreover, the results of Mendelian randomization showed that SMO is inversely related to the trend of OP and acts as a protective factor; the increase in SMO content reduces the risk of OP.

ABCC1 is a transporter located on the cell membrane, belonging to the ATP‐binding cassette (ABC) transporter family. Due to its transport function, ABCC1 may affect the concentrations of certain signaling molecules or ions inside and outside bone cells, thereby influencing bone formation and remodeling. [38]) found that when the epidermal growth factor receptor (EGFR) signaling is activated, it triggers the differentiation of osteoclasts, which further leads to OP. During this process, the expression level of ABCC1 shows a significant upward trend. The results of KEGG showed that the active ingredients in *Anemarrhena asphodeloides* acted on the EGFR signaling pathway in the treatment of OP. We speculate that they may inhibit the activation of the EGFR signaling pathway, thereby reducing the expression of ABCC1 to achieve the purpose of preventing and treating OP.

## 5. Conclusion

Multiple active components in *Anemarrhena asphodeloides* are closely associated with the proteins encoded by AKR1C1, AKR1C2, ABCC1, SMO, and AKT1. The active components in *Anemarrhena asphodeloides* may inhibit the occurrence of OP by suppressing the expression of AKR1C1, AKR1C2, and ABCC1 genes in the body while also inhibiting OP by promoting the expression of AKT1 and SMO. As databases are dynamically updated, this study only speculates on the molecular mechanisms of *Anemarrhena asphodeloides* in the treatment of OP based on the current big data, and further experimental validation is needed in the future.

## Ethics Statement

The data involved all originate from publicly published GWAS summary databases, which complies with the conditions for exemption from review as stated in the “Ethical Review Measures for Life Sciences and Medical Research Involving Humans.”

## Disclosure

All authors have read and agreed to the published version of the manuscript.

## Conflicts of Interest

The authors declare no conflicts of interest.

## Author Contributions

Conceptualization: Y.W. and P.H.; methodology: Q.W.; software: T.H.; validation: Z.W., Q.W., and T.H.; formal analysis: S.L.; investigation: Y.W. and T.H.; resources: Q.W.; data curation: T.H. and Z.W.; writing original draft preparation: T.H. and S.L.; writing, review and editing: S.L. and P.H.; visualization and supervision: Q.W. and J.W.; project administration, funding acquisition: Q.W. and J.W. T.H. and S.L. contributed equally to this work. T.H. and S.L. are co‐first authors.

## Funding

This work was supported by the Wuhan Health Research Fund (WZ21A06).

## Supporting information


**Supporting Information** Additional supporting information can be found online in the Supporting Information section. The supporting information of this article includes the active components and their targets of *Anemarrhena asphodeloides*, which provides further support for the research conclusion of the main text. The relevant supporting information has been uploaded to the supporting information platform.

## Data Availability

The datasets used and/or analyzed during the current study are available from the corresponding authors on reasonable request.
